# The Effect of Covalently Immobilized FGF-2 on Biphasic Calcium Phosphate Bone Substitute on Enhanced Biological Compatibility and Activity

**DOI:** 10.1155/2015/742192

**Published:** 2015-09-07

**Authors:** Kyung-Suk Moon, Eun-Joo Choi, Seunghan Oh, Sungtae Kim

**Affiliations:** ^1^Department of Dental Biomaterials and Institute of Biomaterial and Implant, College of Dentistry, Wonkwang University, Iksan, Jeonbuk 570-749, Republic of Korea; ^2^Department of Oral and Maxillofacial Surgery, College of Dentistry, Wonkwang University, Iksan, Jeonbuk 570-749, Republic of Korea; ^3^Department of Periodontology, Dental Research Institute, Seoul National University, School of Dentistry, Seoul 110-749, Republic of Korea

## Abstract

The purpose of this research was to covalently graft fibroblast growth factor 2 (FGF-2) onto biphasic calcium phosphate (BCP) via a bifunctional cross-linker technique and to estimate the optimal dose of FGF-2 resulting in the best osteogenic differentiation of human mesenchymal stem cells (hMSCs). SEM observation revealed that the surface of the 100 ng FGF-2 coated BCP was completely covered with the nanoparticles expected to be from the silane coupling agent. XRD, FT-IR, and XPS analysis showed that silane treatment, bifunctional cross-linker coating, and FGF-2 covalent grafts were conducted successfully without deforming the crystalline structure of BCP. An MTT assay demonstrated that FGF-2 coated BCP had good biocompatibility, regardless of the concentration of FGF-2, after 24 or 48 h of incubation. An alkaline phosphatase (ALP) activity assay (14 days of incubation) and the ALP gene expression level of real-time PCR analysis (7 days of incubation) revealed that 50, 100, and 200 ng FGF-2 coated BCP induced the highest activities among all experimental groups and control group (*P* < 0.05). Thus, low concentrations of FGF-2 facilitated excellent osteogenesis and were effective at enhancing osteogenic potential. Also, the bifunctional cross-linker technique is expected to be a more feasible way to induce osteogenic differentiation while minimizing the risk of FGF-2 overdose.

## 1. Introduction

Fibroblast growth factors (FGFs) are known to play critical roles in bone development and early osteogenesis. FGF signaling regulates the expression of various genes related to bone formation and is involved in osteoprogenitor proliferation and differentiation into bone forming cells [1–4]. Among the FGF ligands, fibroblast growth factor-2 (FGF-2) is known to promote the attachment and proliferation of osteoblasts [5, 6]. Many researchers have used various substrates to use FGF-2 to promote osteogenesis* in vitro *and* in vivo*. These substrates include polymer scaffolds, cross-linked gels, and biodegradable hydrogels [1, 7–10].

The concentration of FGF-2 and the application method are thought to directly affect the osteogenesis of osteoblasts* in vitro *and* in vivo* after FGF-2 administration. Several reports documented various results regarding the effect of FGF-2 on osteogenic differentiation [11–14]. Most of these studies were mainly aimed at analyzing the synergic effect of FGF-2 and other compounds or growth factors on osteogenic differentiation. However, the administration type and the amount of FGF-2 influencing the osteogenesis of bone forming cells was not considered. In particular, systematic injections of low concentrations of FGF-2 are thought to expect the ambiguous effect of osteogenesis within a short period and cause the accumulated overdose of growth factor.

Recent studies on the use of FGF-2 in combination with biomaterials, such as biodegradable polymers and calcium phosphate ceramics, reported the promotion of sustained release of FGF-2 and the localized effect of the FGF-2/biomaterial composites [15–18]. However, physical entrapment and coprecipitation techniques used by previous researchers have a possibility of generating mechanical weakness between FGF-2 and the substrate [19, 20]. This may affect the release of FGF-2, which is dependent on the durability of the biomaterials. On the contrary, covalent grafts are expected to be suitable for creating a strong bond between low amounts of FGF-2 and the substrate, regardless of the type of biomaterial. Most studies related to FGF-2 coated hydroxyapatite have been used the technique of physical adsorption, entrapment and coprecipitation to immobilize FGF-2 on hydroxyapatite or calcium phosphate scaffold. However, only a few researches of the FGF-2 coated biomaterials via covalent bonding technique have been published [21].

Thus, the purpose of this study was to prepare and characterize FGF-2 coated biphasic calcium phosphate (BCP) via the bifunctional cross-link method, which is one of covalent bonding techniques. In addition, we sought to find the minimal concentration of FGF-2 that induces excellent osteogenic differentiation and no cytotoxicity in human mesenchymal stem cells (hMSCs).

## 2. Materials and Methods

### 2.1. FGF-2 Grafting Process

**Figure 1:**
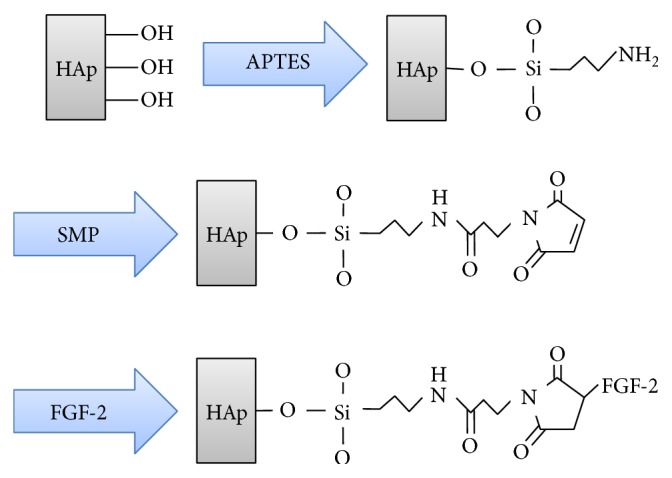
Schematic diagram of the surface modification procedure of biphasic calcium phosphate (BCP): (I) APTES treatment; (II) Bi-functional cross-linker (SMP) connection; (III) FGF-2 covalent immobilization.

The biphasic calcium phosphate (BCP) granule (Osteon GBC0305,) was obtained from Dentium Corporation, Suwon, Korea, and FGF-2 was obtained from the Genoss Corporation, Suwon, Korea, respectively. BCP is a 70% hydroxyapatite (HAp) scaffold coated with 30% *β*-tricalcium phosphate (*β*-TCP) with a particle size of 0.3–0.5 *μ*m. As previously reported [22], the procedure for preparing FGF-2 coated BCP (see Figure 1) includes the grafting of 3-aminopropyl triethoxysilane (APTES, Sigma, MO, USA) onto the BCP surface and the replacement of the terminal amine by a maleimide functional group that reacts with FGF-2 via a heterobifunctional cross-linker (N-Succinimidyl-3-maleimidopropionate: SMP, Sigma, MO, USA). Briefly, 1.0 g of BCP powder was silanized by 10 mM APTES dissolved in hexane (Sigma, MO, USA) for 2 h. The silanized BCP powder was substituted for maleimide groups by 2 mM SMP dissolved in anhydrous dimethylformamide (DMF, Sigma, MO, USA) for 2 h. Then, FGF-2 dissolved in anhydrous DMF was immobilized on BCP by stirring for 2 h. All experimental procedures were performed at 25°C to avoid the risk of FGF-2 denaturation.

### 2.2. Surface Analysis

The surface morphology of the BCP powder was observed by field emission scanning electron microscopy (FE-SEM; S4800, Hitachi/Horiba Co., Japan). In addition, X-ray diffractometer (X'Pert PRO MRD, PANalytical B.V., Netherlands) with Ni-filtered Cu-K*α* ray, Fourier transform infrared spectroscopy (FT-IR; Nicolet, Thermo Co., WI, USA), and X-ray photoelectron spectroscopy (XPS, K-Alpha ESKA system; Thermo, USA) were used to analyze the changes in chemical composition of the BCP before and after FGF-2 immobilization. For XRD measurement, we fixed the glancing angle of the specimen at 5° against the incident beam, enabling the detection of XRD patterns to be at a depth of less than 5 *μ*m from the top surface of the substrate.

### 2.3. hMSC Culture

We obtained human mesenchymal stem cells (hMSCs) from the Lonza Corporation (Poietics hMSCs, Switzerland). The cell growth media used in this study was composed of *α*-MEM (Invitrogen, CA, USA), 10% fetal bovine serum (FBS) (Invitrogen), and 1% penicillin-streptomycin (Invitrogen). The hMSCs were incubated at 37°C and 5% atmospheric CO_2_. During the study, the hMSCs were passaged 4-5 times. After confluence, they were seeded onto BCP powder, placed in a 24-well plate (cell density of 10,000 cells per well), and were stored in a CO_2_ incubator for a range of incubation times. Two days after hMSC seeding, osteogenic induction media consisting of 10 mM *β*-glycerol phosphate (Sigma, MO, USA), 150 *μ*g/mL ascorbic acid (Sigma, MO, USA), and 10 nM dexamethasone (Sigma, MO, USA) was added to promote osteogenic differentiation. The osteogenic induction medium was changed every 2 days.

### 2.4. Cell Adhesion and Proliferation Test

To test the degree of cell adhesion at the beginning of the incubation time, fluorescein diacetate (FDA; Sigma, MO, USA) staining was conducted to visualize viable hMSCs adhered to the experimental specimen. At 24 and 48 h after plating, FDA staining was performed. The detailed procedure of FDA staining was previously reported [23]. The FDA stained specimens were viewed under an inverted fluorescence microscope (CKX41, Olympus Co., Japan). An MTT (3-(4,5-dimethylthiazol-2-yl)-2,5-diphenyltetrazolium bromide) assay was conducted to investigate the proliferation of hMSCs cultured on various experimental specimens. 1 mL of MTT dye agent (Sigma, MO, USA) was added to each well. After 3 h of incubation, 1 mL of isopropanol (Sigma, MO, USA) was added to each well, and the 24-well plate was then shaken for 30 minutes. The absorbance of each solution was measured at 570 nm by a microplate ELISA reader (SpectraMax 250, Thermo Electron Co., USA).

### 2.5. Alkaline Phosphatase (ALP) Activity Assay

After 7 and 14 days of incubation, the experimental samples were rinsed with PBS solution, lysed by lysis buffer solution (25 mM Tris, pH 7.6, 150 mM NaCl, and 1% NP-40) and stored in ice for 30 min. Cell lysate (50 *μ*L) was used for the ALP assay, and the rest of the cell lysate was used for the measurement of the total protein concentration (Bradford Protein Assay Kit, Bio-Rad Laboratories, USA). Cell lysate (50 *μ*L) was mixed with 200 *μ*L of paranitrophenylphosphate (p-NPP, Sigma, MO, USA), and the mixed solution was stored at 37°C for 30 min to activate the reaction. After 30 min, 50 *μ*L of 3 N NaOH (Sigma, MO, USA) was added to the mixed solution to stop the reaction. The absorbance of the mixed solution was measured at 405 nm by a microplate ELISA reader (Spectra Max 250, Molecular Device, CA, USA). The level of ALP activity was normalized to the amounts of total protein in the cell lysates (units/mg protein).

### 2.6. Real-Time PCR Assay [23]

After 7 days of culture, total RNA of the cells cultured on samples was extracted by TRI agent (Invitrogen, USA), and then the extract was reverse-transcribed into cDNA by cDNA Synthesis Kit (SuperScript VILO; Invitrogen, USA). SYBR Green Dye-Based Gene Expression Assays (Invitrogen, USA) was used to perform real-time PCR (StepOne Real-Time PCR System, Applied Biosystems, USA) assay. The detailed information of PCR primer used in this study is listed in Table 1. cDNA samples (1 *μ*L for total volume of 20 *μ*L) were analyzed for interested genes and for house-keeping gene GAPDH. To quantify the gene expression level of each sample, cycle-threshold point was compared by analysis software assisted with real-time PCR. All levels of expression of experimental group were normalized by the level of expression of control group (hMSCs cultured on pure BCP without osteogenic induction media).

### 2.7. Data Analysis

The data of MTT assay and ALP activity assay were expressed as mean ± standard deviation and analyzed statistically by one-way ANOVA (SPSS 12.0; SPSS GmbH, Germany) and post hoc Duncan's multiple range tests. The data of real-time PCR assay were analyzed statistically by paired *t*-test. Differences were considered significant if *P* values were less than 0.05.

## 3. Results and Discussion

### 3.1. Surface Characterization of FGF-2 Coated onto BCP

**Figure 2:**
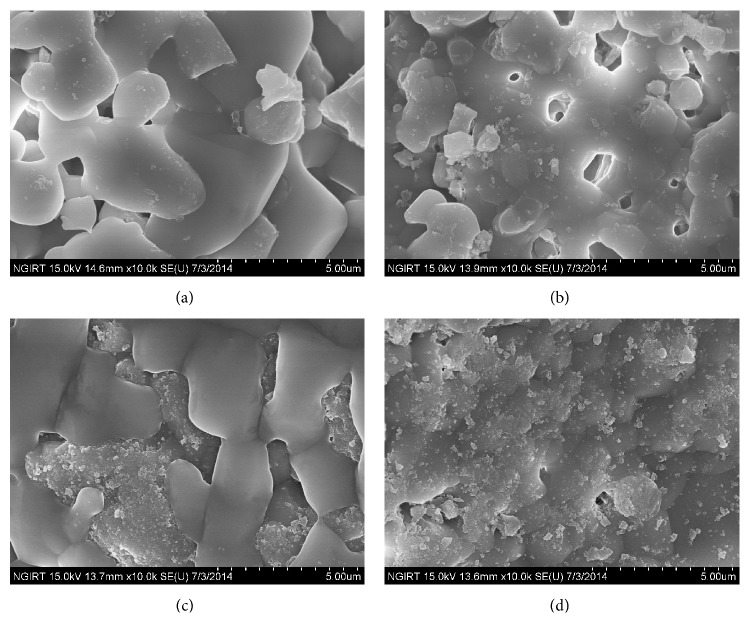
SEM images of (a) BCP, (b) silanized BCP, (c) 50 ng FGF-2-coated BCP, and (d) 100 ng FGF-2 coated BCP.

**Figure 3:**
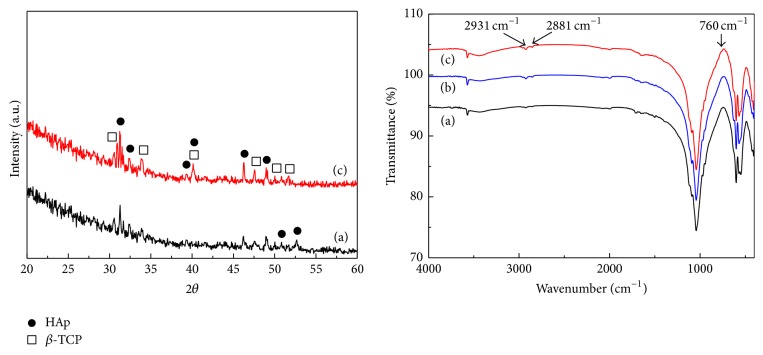
XRD patterns and FT-IR spectra of (a) BCP, (b) silanized BCP, and (c) 100 ng FGF-2 coated BCP.

Figure 2 shows SEM images of BCP, silanized BCP, and 50 and 100 ng FGF-2 coated BCP, respectively. As shown in Figure 2, 10–20 nm nanoparticles were deposited on and partially covered the 50 ng FGF-2 coated BCP. However, they covered the whole surface of 100 ng FGF-2 coated BCP.  Figure 3 indicates the XRD patterns and FT-IR spectra of the BCP, silanized BCP, and 100 ng FGF-2 coated BCP, respectively. As shown by the XRD patterns, the HAp and *β*-TCP crystalline structures were confirmed [24, 25], and there was no structural change after the silane treatment and the FGF-2 grafting process. In the FT-IR spectra, new peaks corresponding to the silane treatment of APTES were detected at 760, 2881, and 2931 cm^−1^ in the FT-IR spectra of the silanized and FGF-2 coated BCP [26, 27]. These peaks are reported to result from the formation of SiO_3/2_ (silsesquioxane) nanoparticles [28].

**Figure 4:**
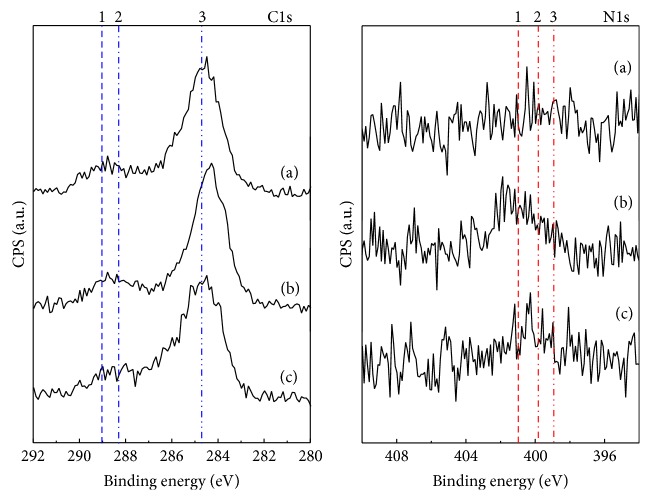
XPS, C1s, and N1s spectra of (a) silanized BCP, (b) bifunctional cross-linked (maleimide) BCP, and (c) 100 ng FGF-2 coated BCP.

Figure 4 shows the XPS spectra of C1s and N1s for silanized BCP, bifunctional cross-linked BCP, and 100 ng FGF-2 coated BCP, respectively. From the C1s spectra, four peaks (lines 1, 2, 3, and 4) were detected from experimental specimens as shown in Figure 4. Lines 1 and 2 (detected at 289 and 288.3 eV, resp.) indicate the chemical structures of O=C-O and C=O. These peaks are thought to originate from maleimide (bifunctional cross-linker) and FGF-2. Line 3 (284.8 eV) represents the silanization of BCP via APTES treatment [29]. In the N1s XPS spectra, 3 peaks were detected on experimental samples. Line 1 (399.7–400.7 eV) shows the existence of maleimide, and line 2 (399.7–400.8 eV) is the portion of N-C=O originating from maleimide and FGF-2. Line 3 (398.9 eV) represents the chemical structures of C-NH_2_ and C-O and is thought to originate from FGF-2.

From the results of SEM observation and FT-IR analysis, 10–20 nm SiO_3/2_ nanoparticles were deposited on the whole surface of the 100 ng FGF-2 coated BCP. XRD and XPS analyses indicated that FGF-2 was coated onto the surface of the BCP through silanization and bifunctional cross-linking without causing structural deformation of the BCP. It is well known that there are free thiol groups on FGF2 existing outside and activating binding site [30–32]. Particularly, FGF-2 has four cysteine residues binding to the maleimide groups of bifunctional cross-linker used in this study. Thus, FGF-2 is expected be coated onto the surface of BCP on the basis of the complete silanization and bifunctional cross-linking of BCP surface.

### 3.2. Initial Attachment and Proliferation of hMSCs

In terms of biological assay testing, the effect of FGF-2 covalently coated BCP on the osteogenic differentiation of hMSCs, an experimental group performing 20 ng FGF-2 dosed to cell culture media every 2 days was added to evaluate the comparison of the previous results of the effect of FGF-2 injection to the culture media on the osteogenic differentiation of cells [5, 13] to the results of this study. The detailed information of control group and experimental groups are listed in Table 2.

**Figure 5:**
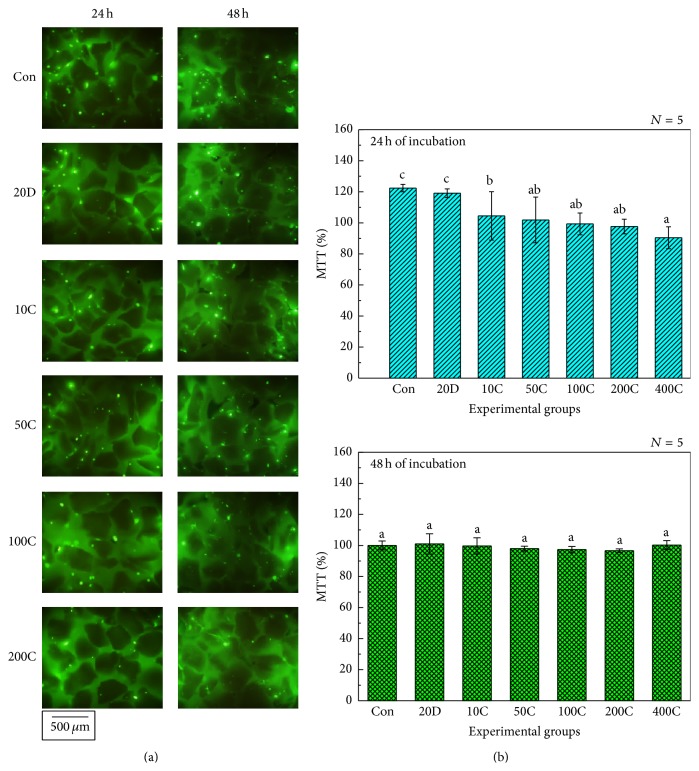
(a) Images of FDA–stained cells and (b) MTT assay results from hMSCs. Con: Uncoated Biphasic Calcium Phosphate (control). 20D: Cells cultured on BCP dosed with 20 ng FGF-2. 10C, 50C, 100C, 200C, and 400C: 10, 50, 100, 200, and 400 ng FGF-2 coated BCP. Different alphabetical letter shows statistical differences.

Figure 5(a) displays the images of FDA-stained live hMSCs cultured on experimental specimens after 24 and 48 h of incubation. Unlike a cell culture dish, BCP powder did not provide a flat surface on which to culture the hMSCs. Because of this, it was difficult to count live hMSCs cultured on BCP powder. Thus, we simply confirmed from the images of the FDA-stained cells that there was no drastic reduction in the number of live hMSCs between the experimental groups regardless of the incubation time.  Figure 5(b) indicates the results of the MTT assay from hMSCs cultured on experimental specimens. After 24 h of incubation, the reduction in MTT values was inversely proportional to the amount of FGF-2 grafted. In addition, the MTT value of the 400 ng FGF-2 coated BCP (400C) was significantly lower than that of the control group, the culture media containing 20 ng FGF-2 (20D), and the 10 ng FGF-2 coated BCP (10C) (*P* < 0.05). However, the MTT values showed that there was no significant difference between experimental groups after 48 h of incubation. According to the ISO 10993-5, if the relative MTT value of specimen is higher than 70%, the specimen has biocompatibility [33]. Thus, it was confirmed that all experimental specimens showed excellent biocompatibility after 24 and 48 hours of incubation and the proliferation of hMSCs was not affected by the amount of coated FGF-2 after 48 h of incubation.

### 3.3. Alkaline Phosphatase (ALP) Activity of hMSCs

**Figure 6:**
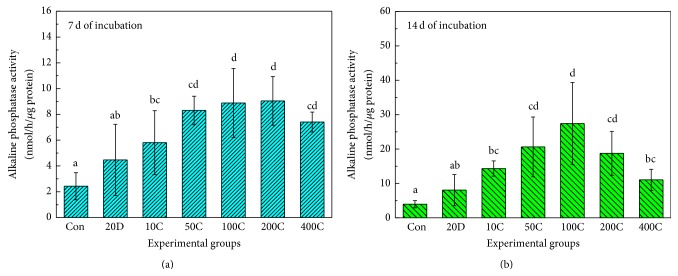
ALP activity assay of hMSCs. Con: Uncoated Biphasic Calcium Phosphate (control). 20D: Cells cultured on BCP dosed with 20 ng FGF-2. 10C, 50C, 100C, 200C, and 400C: 10, 50, 100, 200, and 400 ng FGF-2 coated BCP. Different alphabetical letter shows statistical differences.

Figure 6 illustrates the results of an ALP activity assay on hMSCs cultured on experimental specimens after 7 and 14 days of incubation. As shown in Figure 6(a), the ALP activity of the control group was significantly lower than that of any of the FGF-2 coated BCP groups. The activities of the 50, 100, 200, and 400 ng FGF-2 coated BCP groups (50C, 100C, 200C, and 400C) were significantly higher than that of the group in which 20 ng FGF-2 was added to the culture media (20D) (*P* < 0.05). Thus, it was confirmed that within a short incubation period, covalent grafting of FGF-2 caused higher osteogenic differentiation of hMSCs than when FGF-2 was added to the cell culture media.  Figure 6(b) indicates the ALP activities of hMSCs after 14 days of incubation. As shown in Figure 6(b), 50, 100, and 200 ng FGF-2 coated BCP (50, 100C, and 200C) showed significantly higher values than other experimental groups and the control group (*P* < 0.05). Interestingly, the ALP activities of the 100 ng FGF-2 coated BCP groups showed the trend of the highest values among all experimental groups, even though there is no significant difference among 50, 100, and 200 ng FGF-2 coated BCP groups. In addition, the values of ALP activity have a tendency to be reduced on the condition of more than 100 ng FGF-2 coated BCP groups within this research. From the results of ALP activity, we could expect that covalent bonding of FGF-2 is more effective than addition of FGF-2 to culture media with regard to the osteogenic differentiation of hMSCs. However, the grafting of high concentrations of FGF-2 seemed to inhibit the ALP activity of hMSCs. With regard to the unique trend of ALP activity results, we are currently identifying the factor that prohibits the differentiation of hMSCs into osteoblasts when high concentrations of FGF-2 are coated onto BCP.

### 3.4. Real-Time Assay of hMSCs

**Figure 7:**
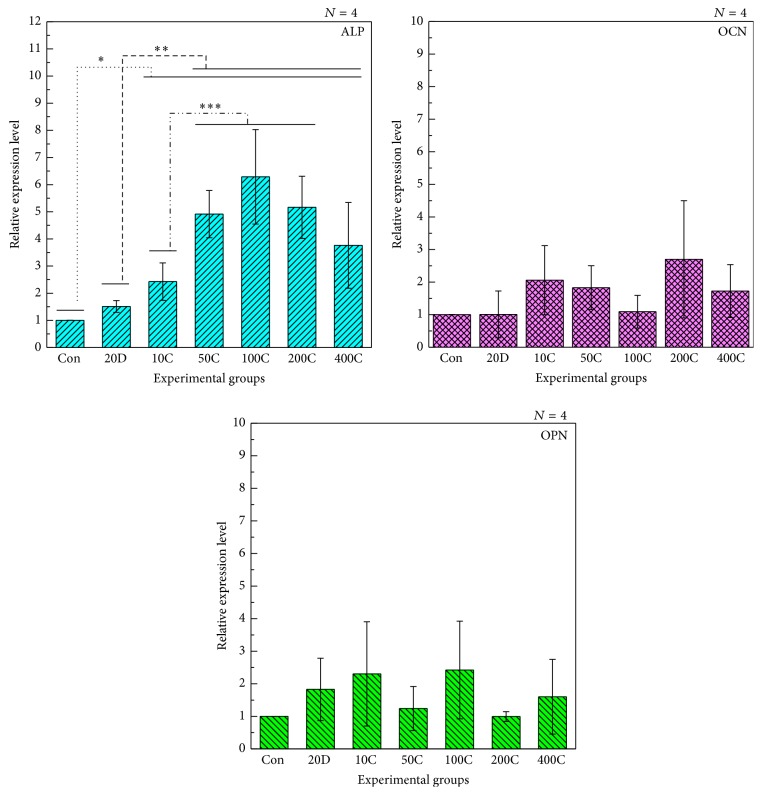
Real time PCR assay (7 days for incubation). Con: Uncoated Biphasic Calcium Phosphate (control). 20D: 20 ng FGF-2 was dosed to cells cultured on BCP. 10C, 50C, 100C, 200C, and 400C: 10, 50, 100, 200, and 400 ng FGF-2 coated BCP.

To assess the osteogenic functionality of experimental specimens, real-time PCR analysis for alkaline phosphatase (ALP), osteocalcin (OCN), and osteopontin (OPN) expression after 7 days of incubation were performed (Figure 7). Expression of OCN and OPN genes was not significantly different between all experimental groups. However, three significant differences between experimental groups were detected from the expression results of ALP gene. Within all groups, the ALP expression value of the 10, 50, 100, 200, and 400 ng FGF-2 coated BCP groups (10C, 50C, 100C, 200C, and 400C) were significantly higher than that of control group. Within all experimental groups excepting control group, the ALP expression value of the 50, 100, 200, and 400 ng FGF-2 coated BCP groups (50C, 100C, 200C, and 400C) were significantly higher than that of the group in which 20 ng FGF-2 was added to the culture media (20D). In addition, within all FGF-2 coated BCP groups, the ALP expression value of the 50, 100, and 200 ng FGF-2 coated BCP groups (50C, 100C, and 200C) was significantly higher than that of the 10 ng FGF-2 coated BCP group (10C). Thus, real-time PCR results were mostly similar to the results of ALP activity assay. In terms of the behavior of OCN and OPN gene expression, ALP gene was already known to be the first expressed phenotypical marker of bone formation and followed by OPN and OCN gene [34, 35]. Thus, the results of real-time PCR assay after 7 days of incubation are supposed to be similar to the behavior of researches previously reported.

Alizarin red S assay was also conducted to evaluate the formation of bone nodule at the surface of BCP. However, alizarin red S solution stains calcium of both newly formed bone nodule and BCP substrate at the same time (data are not shown). Thus, ALP activity assay and real-time PCR analysis were performed to estimate the osteogenesis of experimental groups instead.

### 3.5. Anti-FGF-2 FITC Immunofluorescent Staining

**Figure 8:**
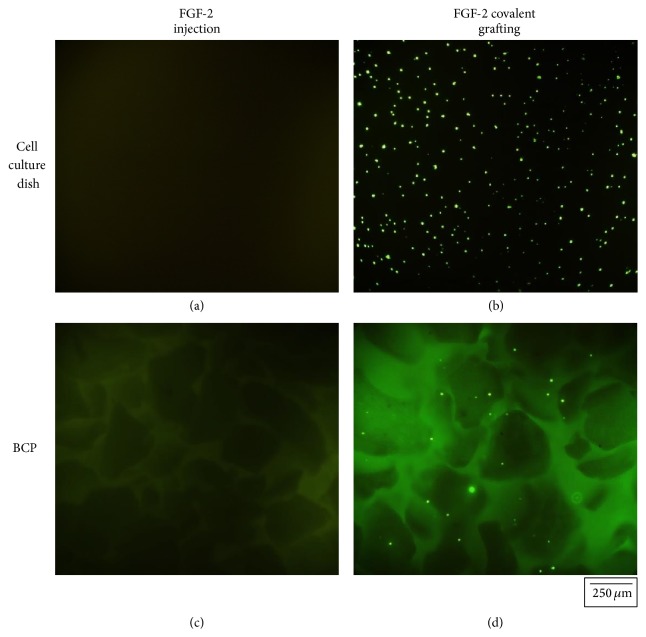
Fluorescent images of anti-FGF-2 FITC staining on experimental specimens; FGF-2 was injected directly to (a) cell culture dish and (c) BCP included cell culture dish, and was grafted covalently onto (b) cell culture dish and (d) BCP by using bifunctional cross-linker.

To confirm that FGF-2 was successfully coated onto the BCP surface, we stained FGF-2 by using a FITC antibody labeling kit (Pierce, Thermo Scientific, Co., USA). FGF-2 was administrated to the surface of a cell culture dish and to BCP by injection and covalent bonding methods, respectively. Then, FITC antibody labeling was performed according to the instruction manual. For detecting FITC antibody-labeled FGF-2, a fluorescent inverted microscope (CKX-41, Olympus, Co., Japan) was used and FITC was visualized under 526 nm green light. Figure 7 illustrates the FITC immunofluorescence images of FGF-2 in cell culture media and coated onto the BCP surface. As shown in Figure 7, FGF-2 covalently coated onto the surface of a cell culture dish or onto BCP was detected clearly by FITC immunofluorescence labeling. In contrast, we could not detect FITC signal in cell culture media dosed with FGF-2 (see Figure 8).

## 4. Conclusion

Fibroblast growth factors are known to be involved in angiogenesis, embryonic development, and bone formation [36–38]. In addition, FGFs are closely related to interact with cell-surface associated reaction and play an important role in the proliferation and differentiation of various cells and tissues. Thus, FGF-2 seems to be related to the interaction between cell and matrix, and this interaction is more effective, if FGF-2 is located between cell and matrix instead of the continuous injection of FGF-2 into the culture media. From the surface characterization, we confirmed that FGF-2 was bonded covalently onto the surface of BCP by the bifunctional cross-linker technique, and 100 ng FGF-2 was enough to cover a whole BCP granule from the images of FE-SEM. The results of the biological assay revealed that high concentrations of FGF-2 reduced the initial attachment of hMSCs to the surface of BCP at the beginning of incubation, but hMSC proliferation was not affected by the amount of coated FGF-2 after 48 h of incubation. In addition, the results of ALP activity assay and the ALP gene expression level of real-time PCR analysis indicated that small amounts of FGF-2 were enough to promote the ALP activity of hMSCs compared to other experimental groups and the control group. Thus, covalent immobilization technique is expected to be useful for enhancing the osteogenesis of bone forming cells by low concentrated growth factors.

## Figures and Tables

**Table 1:** Primer sequences used in real-time PCR.

Target	Sequences
GAPDH	Forward 5′-CAA TGA CCC CTT CAT TGA CC-3′
	Reverse 5′-GAC AAG CTT CCC GTT CTC AG-3′

Alkaline phosphatase (ALP)	Forward 5′-ATC TTT GGT CTG GCC CCC ATG-3′
	Reverse 5′-ATG CAG GCT GCA TAC GCC AT-3′

Osteopontin (OPN)	Forward 5′-AAG CGA GGA GTT GAA TGG-3′
	Reverse 5′-GGA AAG TTC CTG ACT ATC-3′

Osteocalcin (OCN)	Forward 5′-CGC AGC CAC CGA GAC ACC AT-3′
	Reverse 5′-AGG GCA AGG GGA AGA GGA AAG AA-3′

**Table 2:** Detailed information of experimental specimens used in this study.

Designation	Explanation
Con (Control)	Uncoated biphasic calcium phosphate (BCP)

20D	Cell cultured on BCP dosed with 20 ng FGF-2 every 2 days

10C, 50C, 100C, 200C, and 400C	10, 50, 100, 200, and 400 ng FGF-2 covalently coated BCP

## References

[1] Lisignoli G., Zini N., Remiddi G. (2001). Basic fibroblast growth factor enhances in vitro mineralization of rat bone marrow stromal cells grown on non-woven hyaluronic acid based polymer scaffold. *Biomaterials*.

[2] Nakamura T., Hara Y., Tagawa M. (1998). Recombinant human basic fibroblast growth factor accelerates fracture healing by enhancing callus remodeling in experimental dog tibial fracture. *Journal of Bone and Mineral Research*.

[3] Wang J.-S., Aspenberg P. (1996). Basic fibroblast growth factor promotes bone ingrowth in porous hydroxyapatite. *Clinical Orthopaedics and Related Research*.

[4] Zellin G., Linde A. (2000). Effects of recombinant human fibroblast growth factor-2 on osteogenic cell populations during orthopic osteogenesis in vivo. *Bone*.

[5] Marie P. J. (2003). Fibroblast growth factor signaling controlling osteoblast differentiation. *Gene*.

[6] Maegawa N., Kawamura K., Hirose M., Yajima H., Takakura Y., Ohgushi H. (2007). Enhancement of osteoblastic differentiation of mesenchymal stromal cells cultured by selective combination of bone morphogenetic protein-2 (BMP-2) and fibroblast growth factor-2 (FGF-2). *Journal of Tissue Engineering and Regenerative Medicine*.

[7] Tanihara M., Suzuki Y., Yamamoto E., Noguchi A., Mizushima Y. (2001). Sustained release of basic fibroblast growth factor and angiogenesis in a novel covalently crosslinked gel of heparin and alginate. *Journal of Biomedical Materials Research*.

[8] Yamamoto M., Ikada Y., Tabata Y. (2001). Controlled release of growth factors based on biodegradation of gelatin hydrogel. *Journal of Biomaterials Science. Polymer Edition*.

[9] Tabata Y., Ikada Y. (1999). Vascularization effect of basic fibroblast growth factor released from gelatin hydrogels with different biodegradabilities. *Biomaterials*.

[10] Zellin G., Linde A. (2000). Effects of recombinant human fibroblast growth factor-2 on osteogenic cell populations during orthopic osteogenesis in vivo. *Bone*.

[11] Behr B., Sorkin M., Lehnhardt M., Renda A., Longaker M. T., Quarto N. (2012). A comparative analysis of the osteogenic effects of BMP-2, FGF-2, and VEGFA in a calvarial defect model. *Tissue Engineering Part A*.

[12] Quarto N., Longaker M. T. (2006). FGF-2 inhibits osteogenesis in mouse adipose tissue-derived stromal cells and sustains their proliferative and osteogenic potential state. *Tissue Engineering*.

[13] Ito T., Sawada R., Fujiwara Y., Tsuchiya T. (2008). FGF-2 increases osteogenic and chondrogenic differentiation potentials of human mesenchymal stem cells by inactivation of TGF-*β* signaling. *Cytotechnology*.

[14] Su C.-C., Kao C.-T., Hung C.-J., Chen Y.-J., Huang T.-H., Shie M.-Y. (2014). Regulation of physicochemical properties, osteogenesis activity, and fibroblast growth factor-2 release ability of *β*-tricalcium phosphate for bone cement by calcium silicate. *Materials Science and Engineering C*.

[15] Yun Y.-R., Won J. E., Jeon E. (2010). Fibroblast growth factors: biology, function, and application for tissue regeneration. *Journal of Tissue Engineering*.

[16] Lee K., Silva E. A., Mooney D. J. (2011). Growth factor delivery-based tissue engineering: general approaches and a review of recent developments. *Journal of the Royal Society Interface*.

[17] Mima Y., Fukumoto S., Koyama H. (2012). Enhancement of cell-based therapeutic angiogenesis using a novel type of injectable scaffolds of hydroxyapatite-polymer nanocomposite microspheres. *PLoS ONE*.

[18] Ibara A., Miyaji H., Fugetsu B. (2013). Osteoconductivity and biodegradability of collagen scaffold coated with nano-*β*-TCP and fibroblast growth factor 2. *Journal of Nanomaterials*.

[19] Jeong I., Yu H.-S., Kim M.-K., Jang J.-H., Kim H.-W. (2010). FGF2-adsorbed macroporous hydroxyapatite bone granules stimulate in vitro osteoblastic gene expression and differentiation. *Journal of Materials Science: Materials in Medicine*.

[20] Sogo Y., Ito A., Onoguchi M., Oyane A., Tsurushima H., Ichinose N. (2007). Formation of a FGF-2 and calcium phosphate composite layer on a hydroxyapatite ceramic for promoting bone formation. *Biomedical Materials*.

[21] Nur-E-Kamal A., Ahmed I., Kamal J., Babu A. N., Schindler M., Meiners S. (2008). Covalently attached FGF-2 to three-dimensional polyamide nanofibrillar surfaces demonstrates enhanced biological stability and activity. *Molecular and Cellular Biochemistry*.

[22] Oh S., Moon K. S., Lee S. H. (2013). Effect of RGD peptide-coated TiO_2_ nanotubes on the attachment, proliferation, and functionality of bone-related cells. *Journal of Nanomaterials*.

[23] Moon K. S., Yu S. H., Bae J. M., Oh S. (2012). Biphasic osteogenic characteristics of human mesenchymal stem cells cultured on TiO_2_ nanotubes of different diameters. *Journal of Nanomaterials*.

[24] Chou J., Ito T., Bishop D., Otsuka M., Ben-Nissan B., Milthorpe B. (2013). Controlled release of simvastatin from biomimetic *β*-TCP drug delivery system. *PLoS ONE*.

[25] Feng P., Niu M., Gao C., Peng S., Shuai C. (2014). A novel two-step sintering for nano-hydroxyapatite scaffolds for bone tissue engineering. *Scientific Reports*.

[26] Zhang Y., Yuan Y., Liu C. S. (2008). Fluorescent labeling of nanometer hydroxyapatite. *Journal of Materials Science & Technology*.

[27] Vandenberg E. T., Bertilsson L., Liedberg B. (1991). Structure of 3-aminopropyl triethoxy silane on silicon oxide. *Journal of Colloid and Interface Science*.

[28] Lu G., Huang Y., Yan Y., Zhao T., Yu Y. (2003). Synthesis and properties of bismaleimide-modified novolak resin/silsesquioxane nanocomposites. *Journal of Polymer Science A: Polymer Chemistry*.

[29] Durrieu M. C., Pallu S., Guillemot F. (2004). Grafting RGD containing peptides onto hydroxyapatite to promote osteoblastic cells adhesion. *Journal of Materials Science: Materials in Medicine*.

[30] Lee J., Blaber M. (2009). Structural basis of conserved cysteine in the fibroblast growth factor family: evidence for a vestigial half-cystine. *Journal of Molecular Biology*.

[31] Clark R. A. F. (1996). *The Molecular and Cellular Biology of Wound Repair*.

[32] Goldberg I. D., Rosen E. M. (1997). *Regulation of Angiogenesis*.

[33] ISO 10993-5 (2009). *Biological Evaluation of Medical Device—Part 5: Tests for In Vitro Cytotoxicity*.

[34] Stein G. S., Lian J. B. (1993). Molecular mechanisms mediating proliferation/differentiation interrelationships during progressive development of the osteoblast phenotype. *Endocrine Reviews*.

[35] Dalby M. J., Gadegaard N., Tare R. (2007). The control of human mesenchymal cell differentiation using nanoscale symmetry and disorder. *Nature Materials*.

[36] Kühn M. C., Willenberg H. S., Schott M. (2012). Adipocyte-secreted factors increase osteoblast proliferation and the OPG/RANKL ratio to influence osteoclast formation. *Molecular and Cellular Endocrinology*.

[37] Lee T.-J., Jang J., Kang S. (2013). Enhancement of osteogenic and chondrogenic differentiation of human embryonic stem cells by mesodermal lineage induction with BMP-4 and FGF2 treatment. *Biochemical and Biophysical Research Communications*.

[38] Marie P. J., Debiais F., Haÿ E. (2002). Regulation of human cranial osteoblast phenotype by FGF-2, FGFR-2 and BMP-2 signaling. *Histology and Histopathology*.

